# Mechanistic understanding of the toxic effects of arsenic and warfare arsenicals on human health and environment

**DOI:** 10.1007/s10565-022-09710-8

**Published:** 2022-04-01

**Authors:** Suhail Muzaffar, Jasim Khan, Ritesh Srivastava, Marina S. Gorbatyuk, Mohammad Athar

**Affiliations:** 1 UAB Research Center of Excellence in Arsenicals and Department of Dermatology, University of Alabama at Birmingham, Volker Hall - Room 509 1670 University Blvd. , Birmingham, AL 35294-0019 USA; 2 Department of Optometry and Vision Science, The University of Alabama at Birmingham, School of Optometry, Birmingham, AL USA

**Keywords:** Arsenic, Groundwater arsenic, Arsenic in food, Chemical warfare agents, Human risk, Environment

## Abstract

Worldwide, more than 200 million people are estimated to be exposed to unsafe levels of arsenic. Chronic exposure to unsafe levels of groundwater arsenic is responsible for multiple human disorders, including dermal, cardiovascular, neurological, pulmonary, renal, and metabolic conditions. Consumption of rice and seafood (where high levels of arsenic are accumulated) is also responsible for human exposure to arsenic. The toxicity of arsenic compounds varies greatly and may depend on their chemical form, solubility, and concentration. Surprisingly, synthetic organoarsenicals are extremely toxic molecules which created interest in their development as chemical warfare agents (CWAs) during World War I (WWI). Among these CWAs, adamsite, Clark I, Clark II, and lewisite are of critical importance, as stockpiles of these agents still exist worldwide. In addition, unused WWII weaponized arsenicals discarded in water bodies or buried in many parts of the world continue to pose a serious threat to the environment and human health. Metabolic inhibition, oxidative stress, genotoxicity, and epigenetic alterations including micro-RNA-dependent regulation are some of the underlying mechanisms of arsenic toxicity. Mechanistic understanding of the toxicity of organoarsenicals is also critical for the development of effective therapeutic interventions. This review provides comprehensive details and a critical assessment of recently published data on various chemical forms of arsenic, their exposure, and implications on human and environmental health.

**Graphical abstract:**

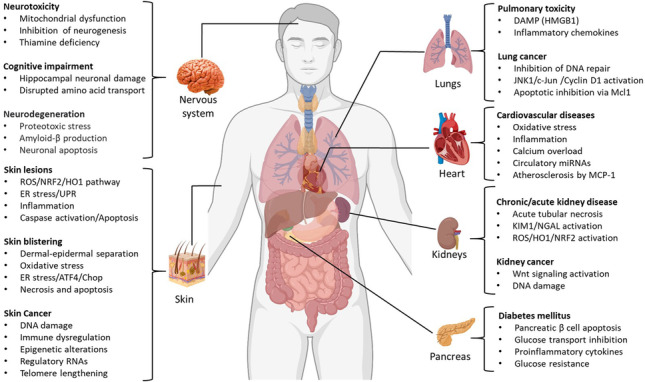

## Introduction

Arsenic is a highly toxic element with no clearly defined essential physiological role in humans. In nature, arsenic exists in different oxidation states and multiple inorganic and organic forms. Inorganic and organic forms of arsenic are converted into each other in the environment. As reviewed by Chen and Costa, arsenic reacts with oxygen and sulfur to form various inorganic compounds, whereas various other chemical and biochemical reactions may generate organic derivatives as well (Chen and Costa, 2021). Also, animals convert inorganic arsenite (AsO_3_^3−^) and arsenate (AsO_4_^3−^) into methylarsonic acid (MMA^V^), dimethylarsinic acid (DMA^V^), monomethylarsonous acid (MMA^III^), and dimethylarsinous acid (DMA^III^) in a series of methylating reactions, as explained later in detail. As reviewed by Rahman et al., the toxic manifestations of arsenic derivatives (arsenicals) depend on their chemical form and biomethylation of inorganic arsenic is generally considered a detoxification process (Rahaman et al., 2021).

Arsenic contamination in groundwater is largely the result of rock erosion, industrial disposal, and agricultural activities. Human exposure to arsenic is a global health concern, as more than half of the world population uses groundwater for drinking, cooking, agricultural, and other household purposes. Arsenic is also present in common dietary sources, including rice and seafood. Rice contains higher levels of inorganic arsenic, whereas seafood predominantly contains organic forms. Due to the absence of color, taste, or odor, it is very difficult to detect arsenic contamination in water, food, or air without proper scientific instrumentation. This leads to the chronic exposure of human populations until the symptoms of toxicity appear. Human exposure to arsenic leads to a variety of health hazards, including skin disorders, cardiovascular disease, diabetes, neurotoxicity, and cancer. However, the skin conditions appear earlier than the other manifestations and often serve as a predictive biomarker of sustained arsenic exposure (Pathania, 2020). Although several population-based studies revealed a strong association between chronic arsenic exposure and human diseases, the mechanistic aspects of underpinning pathogenesis remain poorly defined. In the past, metabolic inhibition, oxidative stress, and genotoxicity were believed to be the underlying mechanisms of arsenic toxicity. However, recently, several epigenetic factors including histone modifications and micro-RNA (miRNA) have been found to play a significant role in arsenic exposure-related disorders.

Unlike the common organic arsenicals generated in the environment from the inorganic forms of arsenic, synthetic organoarsenicals like lewisite (2-chlorovinyldichloroarsine) are extremely toxic (Li et al., 2016). These arsenicals have also been used as chemical warfare agents (CWAs) and their extremely toxic properties were exploited to injure, incapacitate, or kill the enemy personnel and civilian populations. Exposure to arsenic-based CWAs not only has acute toxic manifestations but their long-term effects in humans are also known. Organoarsenical stockpiles in many countries pose a continuous threat of accidental exposure and perhaps terrorism (Radke et al., 2014). Unused arsenicals were buried or dumped into water bodies during and after World War II (WWII). Thus littering CWAs on the ocean floor pose a serious threat to the marine ecosystem as well as to human health. In this review, we have discussed recently published data on distribution, sources of human exposure, and mechanism of toxicity of arsenic and warfare arsenicals. Several review articles published on arsenic toxicity in the last decade have mostly focused on groundwater arsenic toxicity. However, this updated review encompass different sources of arsenic exposure including groundwater arsenic and atmospheric arsenic as well as CWAs and their impact on human health and the ecosystem.

## Sources of arsenic exposure

### Groundwater arsenic

Arsenic is a major component in more than 200 minerals and desorption of these minerals results in groundwater contamination. The groundwater of many countries, including the United States of America (USA), India, Bangladesh, China, and Mexico, is naturally contaminated with high levels of inorganic arsenic. Specifically, groundwater arsenic contamination in Bangladesh presents the largest known poisoning of the human population in history (Raju, 2022). In addition to natural contamination, anthropogenic sources are also responsible for arsenic contamination in groundwater. Exposure to chromated copper arsenate is a concern as it was commonly used as a wood preservative. Although this treatment process has been discontinued, the wash-off from the treated wood has also been a continuous source of arsenic contamination in soil and water bodies (Safa et al., 2020).

**Figure 1:**
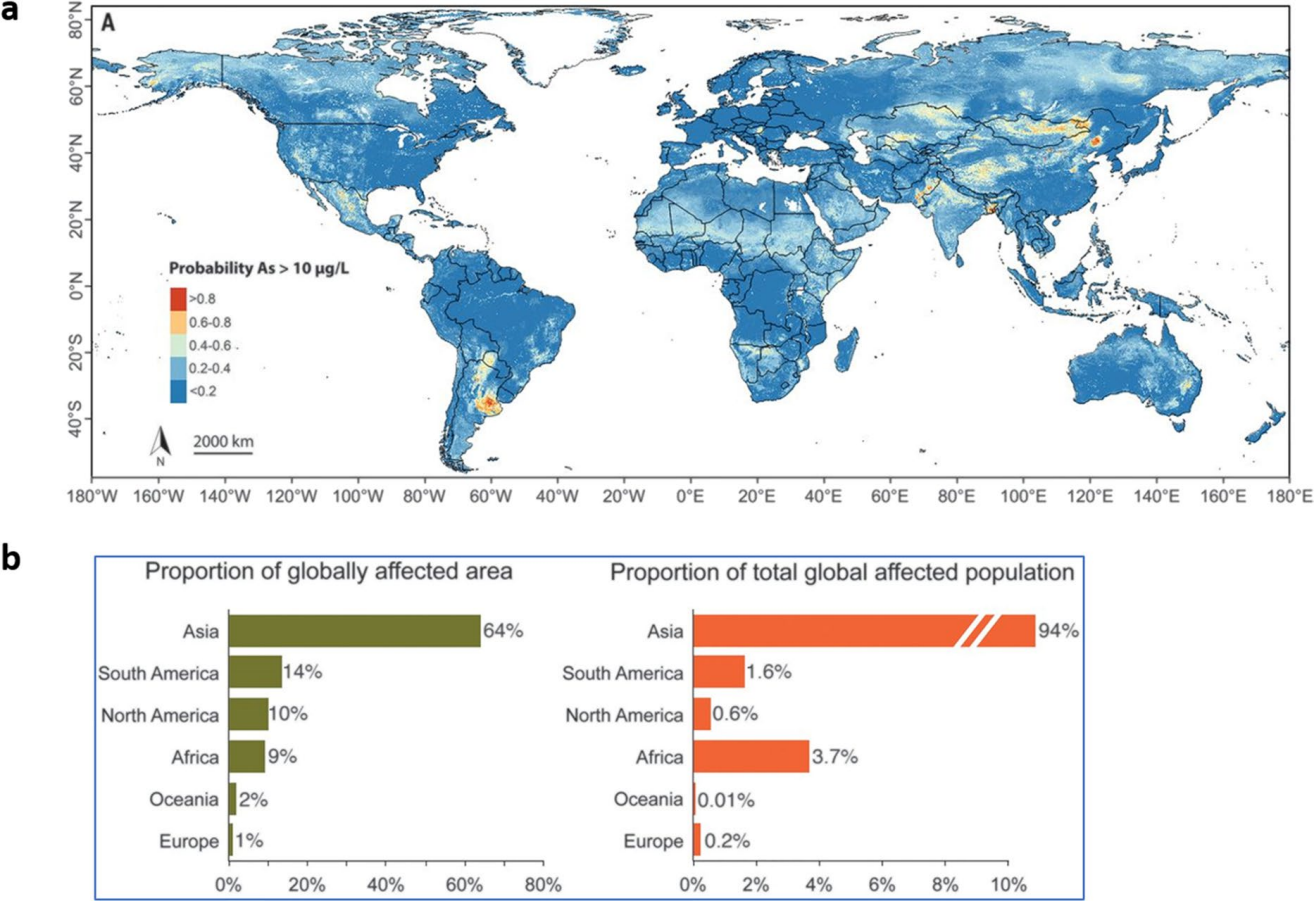
Global groundwater arsenic distribution and the affected populations. **a** World map depicting documented and previously undocumented areas affected by groundwater arsenic contamination. **b** The proportions of land areas, potentially affected populations, and household groundwater usage based on prediction model of the global arsenic (The figure has been adapted from Podgorski and Berg, 2020; License Number 5244990548785)

The World Health Organization (WHO) has prescribed the maximum permissible limits of arsenic in drinking water as 10 μg/L (10 ppb). According to a recent study based on more than 50,000 aggregated data points, it has been estimated that 94 million to 220 million people worldwide are possibly exposed to unacceptably higher levels of groundwater arsenic (Podgorski and Berg, 2020). In this study, Podgorski and Berg used groundwater arsenic data from previous studies for a machine-learning model and predicted groundwater concentrations in the areas where arsenic concentrations were previously undocumented. This study demonstrated that many regions of Central Asia, Southeast Asia, North America, South America, and some parts of Africa have groundwater arsenic concentrations exceeding 10 μg/L (Fig. 1a). However, most cohorts comprising these exposed populations belong to the Asian continent (Fig. 1b). It is well known that groundwater arsenic contamination exceeding WHO limits is a serious health concern in Southeast Asia, in particular. In Bangladesh, almost 40 million people are exposed to arsenic concentrations above 10 μg/L, and about 20 million people are exposed to concentrations above 50 μg/L, which is Bangladesh’s national standard (Loewenberg, 2016). In India, more than ten states have reported more than 10 μg/L arsenic in groundwater, including West Bengal, Assam, and Arunachal Pradesh (Shaji et al., 2021). In addition, the widespread presence of arsenic was reported in the Indus River basin (van Geen et al., 2019). In Pakistan, about 13 million people inhabiting the Indus River region are exposed to arsenic concentrations above 10 µg/L (Rabbani et al., 2017). In China, an area of more than 580,000 km^2^ is at risk of groundwater arsenic contamination (>10 µg/L), and more than 19 million people may be affected as a result (Rodríguez-Lado et al., 2013). Mongolia and Xinjiang provinces are historic hotspots of arsenic contamination where arsenic levels above 500 µg/L have been reported in the drinking water (Deng et al., 2009). The U.S. Environmental Protection Agency (EPA) set the maximum contaminant level (MCL) permissible for arsenic in public water supplies at 10 µg/L. However, more than 44 million people in the USA rely on domestic wells as the principal source of drinking water. According to the U.S. Geological Survey (USGS), 2.1 million Americans in 44 states use water from domestic wells with arsenic levels higher than 10 µg/L (Ayotte et al., 2017). In the US state of Maine, some individual domestic wells contain arsenic levels 50 times higher than the limits set by WHO and the U.S. EPA (Nielsen et al., 2010). A survey of 30,000 groundwater samples in the USA showed that 10% of samples contain arsenic concentrations exceeding 10 µg/L (Welch et al., 2001). Despite huge technological advances and data resources available, groundwater arsenic remains one of the biggest challenges to human civilization today. The lack of sustainable arsenic-free water supply and durable mitigation schemes is evident in most of the affected countries. There is an urgent need for an efficient and cost-effective arsenic detection system in groundwater. Removal of arsenic from drinking water is theoretically an option but doing so at such a large scale is not practical and is not economically sustainable as well. Currently, the best possible prevention and mitigation option to prevent further arsenic exposure in the affected populations is by providing safe and alternative sources of drinking water.

### Atmospheric arsenic

Atmospheric arsenic is emitted due to natural as well as industrial processes including volcanic eruptions, microbial activities, and coal-based power plants. Industrial arsenic emissions lead to air pollution and occupational exposure (Baker et al., 2018). Because of the quick and widespread dispersion, these arsenic emissions may have a huge impact on human health. These volatile arsenic forms may react with atmospheric oxygen and convert back to non-volatile forms before settling back to the ground. Most of the emitted arsenic is deposited onto the surface of particles which are then dispersed by the wind. For example, a study by Atarodi et al. revealed that the residents of Gonabad city in northeast Iran may be exposed to high levels of atmospheric arsenic due to wind-borne arsenic deposition in this area (Atarodi et al., 2018). Accurate assessment of atmospheric arsenic is crucial for the evaluation of health risks in humans. The combined exposure to atmospheric and groundwater arsenic may significantly exacerbate detrimental health effects in humans. However, very limited data exist to evaluate the short- and long-term implications of atmospheric arsenic exposure to humans.

The European Union has established a limit of 6 ng/m^3^ for airborne arsenic; however, various studies have reported arsenic concentrations above these limits in many parts of the world. For example, Chile and China have been hotspots of atmospheric arsenic pollution where airborne arsenic concentrations above 20 ng/m^3^ have been reported (Jiang et al., 2018). However, Zhang et al. reported that the average atmospheric arsenic levels in India have already surpassed the levels of atmospheric arsenic in eastern China, although further studies are needed to support and confirm this notion particularly with current evaluations. From 2005 to 2015, a 65% increase in atmospheric arsenic concentrations has been reported in India, possibly due to massive coal burning. On the other hand, a decrease of 22% in atmospheric arsenic has been reported in eastern China due to strict air quality regulations (Zhang et al., 2020). This shows that ambient air quality standards can be achieved by mitigating emissions from industrial and domestic sources. Indeed, replacing fossil fuel burning with renewable sources of energy will reduce atmospheric arsenic emissions.

### Arsenic in food

The U.S. Food and Drug Administration (FDA) monitors and regulates levels of arsenic in foods. Common food items, particularly rice, seafood, tea, and mushrooms, may contain high levels of arsenic. Worldwide, rice is consumed as a staple food by over half the population. Rice plants bioaccumulate inorganic arsenic at levels approximately ten-fold higher than those in other cereals like wheat and barley (Davis et al., 2017). Reductive dissolution of iron oxyhydroxide minerals and the reduction of arsenic adsorbed on soil particles increase the bioavailability of arsenic to plants (Muehe et al., 2019). The total concentration of arsenic and its bio-accessibility depends on the rice variety, origin, and cooking conditions. For example, brown rice may contain higher levels of arsenic than polished white rice does (Yim et al., 2017). The environmental conditions during plant growth also determine the arsenic content in rice grain, as higher air temperature in the late-ripening stage of rice leads to increased arsenic content (Arao et al., 2018). Higher temperatures (38°C) led to increased inorganic arsenic content in rice grain twofold (Muehe et al., 2019). These observations indicate that arsenic contamination in rice will be magnified in future climatic conditions. Arsenite is the principal form of arsenic in anaerobic paddy soils, ranging between 36 and 63% of the total arsenic (Abedin et al., 2002). Additionally, methylating and reducing microbes present in the soil provide arsenate as MMA^V^ and DMA^V^, which can be detected in rice grain (Muehe et al., 2019). Due to the similarity between arsenite and silicic acid, arsenic is transported into rice plants by silicon transporters. The aquaporin (AQP) channel Lsi1 is responsible for arsenite transport into the roots, whereas Lsi1 and Lsi2 channels transport arsenic in rice shoots and grain (Ma et al., 2008). Compared to inorganic arsenic, methylated arsenic species, including DMA^V^ and MMA^V^, are taken up sluggishly by rice plants but are translocated very efficiently to the rice grain (Carey et al., 2011).

Karagas recently reviewed the correlation between rice consumption and human disorders such as diabetes, cardiovascular diseases, and bladder cancer (Karagas et al., 2019). In 2019, a Multi-Ethnic Study of Atherosclerosis from six US cities revealed that arsenic concentrations in urine were 82% higher in Chinese and Hispanic participants than in White participants. This higher rate of arsenic exposure is supposedly due to the frequent consumption of rice in these ethnic populations (Jones et al., 2019). Although arsenic-contaminated rice could be considered a human health hazard, the long-term implications of its consumption are not clear yet. Several studies associate rice intake with human disorders; however, there is not enough evidence to determine conclusively whether the findings were consistent. More population-based investigations with molecular data are needed to establish a direct link between rice consumption, arsenic levels, and human diseases.

Similarly, seafood consumption is a major source of arsenic exposure for millions of people worldwide. In the marine ecosystem, prokaryotes and phytoplanktons are mainly responsible for the biotransformation of inorganic arsenic into methylarsenicals, where it is introduced into marine life food chain. The levels of arsenic in marine organisms may vary within the range of 5–100 μg per gram of dry mass, depending on the species and location (Francesconi, 2010). Most marine organisms including fish, algae, lobsters, and sharks store arsenic in less toxic forms such as arsenobetaine and arsenosugars. The exact reason behind the reduced toxic manifestations of these organic forms of arsenic is not understood. However, it is likely that these organic forms are less interactive with the body’s biochemical reactions. In contrast to arsenobetaine, arsenosugars may convert into more toxic forms like DMA (Leffers et al., 2013). In Spain, the higher prevalence of diabetes has been associated with high seafood intake and consequent arsenic exposure. Specifically, a study involving 1451 Spanish adults showed a positive association between increased levels of total urinary arsenic, seafood intake, and the prevalence of type 2 diabetes (Grau-Perez et al., 2018). This study indicates that specific genotypes (e.g., single nucleotide polymorphisms related to metabolic disorders) are more susceptible to arsenic-related diabetes; however, the genetic and biochemical evidence supporting this assumption is still lacking.

## Cellular uptake and impact on energy metabolism

When ingested, 70%–90% of inorganic arsenic absorbed by the gastrointestinal tract is distributed to the kidneys, liver, muscles, and nerve tissue (Palma-Lara et al., 2020). Due to similarities in chemical structure with phosphate, arsenate is transported into the cells primarily by the phosphate uptake system, whereas inorganic arsenite ions are largely taken up via aquaglyceroporins or sugar permeases (Garbinski et al., 2019). Aquaglyceroporins are a subfamily of AQPs involved in the transport of glycerol and water from plasma to body organs and vice versa. AQPs are expressed in all major organs of the body, including the liver, lung, spleen, kidney, and adipose tissues. It has been reported that mammalian AQP3, AQP7, and AQP9 are involved in the uptake of inorganic arsenite, while AQP9 also transports MMA^III^ across the cell membrane (Liu et al., 2002).

As previously reviewed by Shen et al., arsenic and its derivatives inhibit about 200 enzymes involved in various biological pathways (Shen et al., 2013). Enzymes involved in the energy metabolism and ATP production are the target of arsenic. For example, arsenate may replace phosphate groups needed for generating pyruvate and ATP during glycolysis. Human proteome microarray analysis revealed that arsenic specifically binds with 360 different proteins and many of these proteins belong to the glycolytic pathway such as hexokinase-1 (HK1) and hexokinase-2 (HK2) (Zhang et al., 2015). Since the enhanced glycolytic pathway is a common characteristic feature of various cancers, the HK2 inhibitory role of arsenic trioxide (ATO) may also provide a possible explanation for its anticancer activities besides its toxicity to normal cells.

In the Krebs cycle, arsenite inhibits the pyruvate dehydrogenase complex (PDH) and α-ketoglutarate dehydrogenase complex (KGDH), thereby uncoupling mitochondrial respiration and ATP synthesis (Bergquist et al., 2009). Specifically, arsenite blocks the regeneration of dihydrolipoamide, an essential component of PDH, and inhibits the conversion of pyruvate to acetyl-CoA. The mammalian mitochondrial electron transport chain generates ATP during oxidative phosphorylation via transmembrane protein complexes I-IV. MMA^III^ targets electron transport by inhibiting the activity of complexes II and IV, resulting in the generation of ROS inside the mitochondria and thus leading to mitochondrial dysfunction. In addition to inhibiting glucose metabolism, arsenic may also disrupt glucose transport. Recently, it was revealed that arsenite causes the degradation of glucose transporters in yeast through a process mediated by E2 ubiquitin ligase Ubc4 and E3 ubiquitin ligase Rsp5 (Jochem et al., 2019). However, it remains to be demonstrated if a similar mechanism also operates in the experimental animal models of human exposure.

## Oxidative metabolism, DNA damage repair, and epigenetic alterations

**Figure 2:**
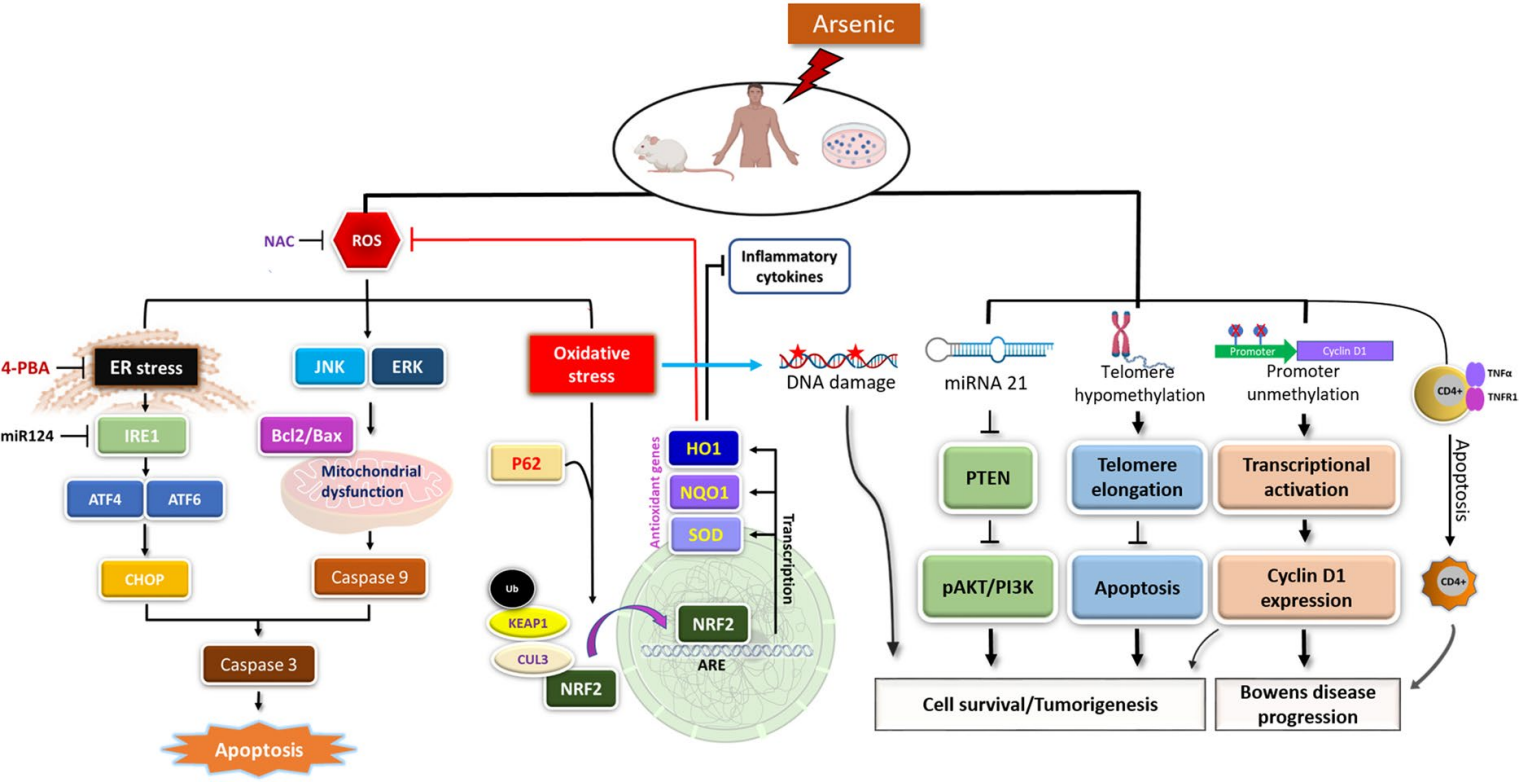
Flow diagram representing arsenic-induced UPR, MAPK, and NRF2 signaling in skin. Arsenic induces accumulation of unfolded proteins in the ER, leading to ER stress in the skin. ER stress activates PERK/ATF4/CHOP signaling and consequently results in apoptotic cell death. Chemical chaperone 4-phenylbutyric acid (PBA) attenuates arsenic-induced ER stress. MAP kinases JNK and ERK1/2 also play a significant role in arsenic-induced inflammation and cell death in human keratinocytes. Arsenic-induced ROS activates the KEAP1/Nrf2/ARE response signaling pathway leading to Nrf2 translocation into the nucleus and thereby augments the transcription of antioxidant genes, e.g., superoxide dismutase (SOD), NADPH quinone dehydrogenase 1 (Nqo1) and heme oxygenase-1 (HO-1). Oxidative stress-mediated chromosomal and mitochondrial DNA mutations could be associated with arsenic-induced skin malignancies. MicroRNAs such as miR-21 could also support the development of arsenic-induced skin lesions. Telomere lengthening is one of the major contributors to cell proliferation, and arsenic promotes telomere lengthening via H4K20me3 hypomethylation. Hypomethylation of oncogenes has been frequently linked with chronic arsenic exposure (This Figure was created using BioRender.com)

In addition to inhibition of energy metabolism, DNA damage and repair, and epigenetic alterations are also key to understanding arsenic-induced toxicity (Table 1). Oxidative stress induced by arsenic and its derivatives results in the activation of the nuclear factor erythroid 2-related factor 2 (NRF2) antioxidant signaling pathway (Fig. 2). NRF2 is a redox-sensitive transcription factor that augments the cellular defenses in response to elevated oxidative stress by arsenic. Keap1, a redox-regulated adaptor protein of the Cul3-dependent ubiquitin ligase complex, senses oxidative stress and halts NRF2 degradation, facilitating NRF2 activation (Lau et al., 2013). The Nrf2/hemeoxygenase-1 (HO1) pathway is activated in response to inorganic arsenic exposure in several cells types, including HaCaT keratinocytes, human hepatocytes, and primary cultured osteoblasts (Chiu et al., 2016; Choi, 2019; Liu et al., 2013). NRF2 confers protection against arsenic toxicity by augmenting the expression of antioxidant enzymes. However, sustained activation of NRF2 may lead to cancer development by contributing to apoptotic resistance and enhanced survival of cells carrying oncogenic mutations (Niture and Jaiswal, 2012). Loss of NRF2 function in Nrf2^−/−^ mice has been reported to enhance arsenic-induced osteoclast differentiation and aggravated bone loss (Liu et al., 2019).

**Table 1:** Arsenic exposure causes various human disorders. Summary of skin lesions, cardiovascular diseases, metabolic disorders, neurotoxicity, and nephrotoxicity and their underlying molecular mechanisms

Skin disorders	Disorder	Arsenic species/source	Mechanism of toxicity	Reference
	Cutaneous toxicity and inflammation	Sodium arsenite	Oxidative stress and UPR signaling	(Li et al., 2011)
		Sodium arsenite	AIM2 inflammasome and IL-1β/IL-18 cytokines	(Zhang et al., 2016)
	Skin hyperpigmentation	Sodium arsenite	Endothelin-1 expression via NF-kappa B activation	(Yajima et al., 2017)
	Skin disorders Skin cancers (Bowens disease/SCC/BCC)	Drinking water arsenic	DNA damage and inhibition of DNA repair	(Muenyi and Ljungman, 2015)
		Drinking water arsenic	Chromosomal aberrations	(Mahata et al., 2003)
		Drinking water arsenic	P53 mutations	(Hsu et al., 1999)
		Inorganic arsenic	Dedifferentiation and generation of cancer stem cells	(Li et al., 2019)
		Drinking water arsenic	Telomere lengthening	(Bhattacharjee et al., 2020)
		Arsenic smelting plant	LncRNA (HOTAIR and LncRNA-p21)	(Tan et al., 2021)
		Drinking water arsenic	Micro RNAs (miRNA21, miR-425-5p, miR-433, miR-184, and miR-576-3p)	(Banerjee et al., 2017); (Al-Eryani et al., 2018)
		Environment	Cyclin D1 promoter unmethylation	(Liao et al., 2018)
		Drinking water arsenic	Immune dysfunction	(Yu et al., 2018)
	Skin vesication	Lewisite (Warfare agent)	ROS, UPR signaling, and apoptosis	(Li et al., 2016)
Cardiovascular disorders	Cardiotoxicity	Sodium arsenite	Cardiac tissue remodeling and inflammation	(Souza et al., 2020)
		Arsenic trioxide	Calcium signaling dysregulation	(Vineetha and Raghu, 2019)
		Arsenic trioxide	ROS, DNA damage, and apoptosis	(Zhao et al., 2008)
		Drinking water arsenic	Micro RNAs (miR-423-5p and miR-454-5p)	(Beck et al., 2018)
	Atherosclerosis	Methylated arsenic	As3MT catalyzed methylation of inorganic arsenic	(Negro Silva et al., 2017)
Metabolic disorders	Diabetes and insulin resistance	Arsenic trioxide	Pancreatic B cell apoptosis	(Lu et al., 2011)
		Sodium arsenite	Micro RNAs (miR-146) and CAMK2A	(Beck et al., 2019)
	Glucose metabolism	Arsenic trioxide	Inhibition of glycolysis, Krebs’s cycle, and ATP synthesis	(Kulshrestha et al., 2014)
	Glucose transport	Sodium arsenite	Inhibition of GLUT4 translocation	(Li et al., 2021)
Nervous system disorders	Neurotoxicity	Sodium arsenite	Decreased activity of mitochondrial complexes	(Prakash and Kumar, 2016)
		Sodium arsenite	Neuronal apoptosis via p38 MAP kinase and JNK3 pathways	(Namgung and Xia, 2001)
		Sodium arsenite	ER stress and miRNA dysregulation	(Park et al., 2020)
	Neurodegeneration	Sodium arsenite	Enhanced amyloid-β production and β-secretase activity	(Niño et al., 2017)
		Sodium arsenite	Proteotoxic stress via interaction with ZNF598 sensor protein	(Tam et al., 2020a, b)
Kidney disorders	Nephrotoxicity	Sodium arsenite	Epigenetic changes (DNA methylation)	(Chang and Singh, 2019b)
		Drinking water arsenic	Kidney Injury Molecule-1 (KIM1)	(Cárdenas-González et al., 2016)
	Kidney cancer	Sodium arsenite	Wnt β-catenin/c-myc pathway	(Chang and Singh, 2019a)
Pulmonary diseases	Lung injury	Environmental arsenic	Mitochondrial and immune dysfunction	(Wang et al., 2020)
		Lewisite (Warfare agent)	Damage-associated molecular pattern molecules	(Manzoor et al., 2020)
	Pulmonary fibrosis	Sodium arsenite	LncRNA H1- mediated M2 polarization of macrophages	(Xiao et al., 2021)
	Lung carcinoma	Environmental arsenic/sodium arsenite	Genetic and epigenetic changes (DNA methylation)	(Martinez et al., 2010); (van Breda et al., 2015)

Trivalent arsenic, including inorganic arsenite and its methylated metabolic forms, inhibits DNA repair by elevating the reactive oxygen species (ROS) and reactive nitrogen species, which may directly perturb DNA repair machinery by modifying cysteine residues in proteins. Arsenic-mediated dysregulation of DNA damage response signaling through protein ubiquitination and SUMOylation also disrupts DNA repair (Tam et al., 2020b). Inability to repair the damaged DNA may trigger the onset of apoptosis in these cells. In addition to apoptosis, arsenic has also been associated with cellular senescence. Recently, ATO was found to induce senescence of human articular chondrocytes and rat articular cartilage via activation of p38 MAP kinases and the upregulation of the senescence-associated proteins p16, p21, and p53. This induction of chondrocyte senescence suggests the role of chronic arsenic exposure in articular cartilage abrasion (Chung et al., 2020).

Epigenetic alterations like DNA methylation and histone modifications have been associated with arsenic toxicity. Based on cord blood-derived DNA from 134 human infants, it has been shown that arsenic exposure may alter the fetal epigenome (Koestler et al., 2013). Also, epigenetic changes such as DNA methylation and histone modifications have been shown to transmit to later generations via sperm from the arsenic-exposed males (Nohara et al., 2020). A study investigating the DNA methylation patterns in arsenic exposed population in Mexico identified 183 genes with differentially methylated promoters. As reviewed by Bailey and Fry, many of these genes with hypermethylated promoters have been associated with arsenic-induced disorders such as cancer, diabetes, and cardiovascular diseases (Bailey and Fry, 2014). A multi-generational family study in Inner Mongolia revealed that arsenic exposure may result in stable DNA methylation patterns that can be observed even decades after the exposure. Interestingly, arsenic exposure across generations shared common methylated DNA loci despite different exposure timings in each generation (Guo et al., 2018). These changes in global DNA methylation patterns were more notable in patients afflicted with arsenic-induced skin lesions. The inheritance of epigenetic changes in arsenic exposed individuals provides novel avenues of scientific research in public health.

In addition to DNA methylation, arsenic has also been linked to post-translational histone modifications such as histone 3 (H3) and histone 4 (H4) methylation. Enhanced acetylation (ac) of histones is normally associated with transcriptional activation; however, the functional significance of methylation (me) depends on various factors. For example, H3K4me2 and H3K4me3 are associated with open chromatin and transcriptional activation; on the contrary, H3K9me3 is related to transcriptional repression. As previously reviewed by Eckstein et al., exposure to inorganic arsenic may lead to global changes in posttranslational histone modifications including H3K9me2, H3K4me3, and H3K27me3 (Eckstein et al., 2017). Pournara et al. reported a significant reduction in global H3K9me3 in CD4+ cells from individuals exposed to arsenic through drinking water in the Argentinean Andes (Pournara et al., 2016). Another study showed an arsenic-induced increase in H3K18ac and a decrease in H4K8ac in the leukocytes collected from individuals exposed to higher concentrations of arsenic (Ge et al., 2018). However, mere changes in global histone acetylation and methylation patterns may not be sufficient to understand their mechanistic role in arsenic toxicity and carcinogenesis. The genomic location of these altered histone marks is crucial for unraveling changes in gene expression and their consequent role in human disorders.

## The impact on the immune system

Epidemiological and experimental studies show that arsenic adversely affects the immune system and leads to immunotoxicity. The immunotoxic effects of arsenic could be related to apoptotic cell death in B cells, T cells, neutrophils, and macrophages, as reviewed by Dangleben et al. (Dangleben et al., 2013). Zarei et al. showed that arsenic-induced apoptosis of lymphocytes is the result of caspase activation, and stimulation of cytokines such as IL2, INF-gamma, and TNF-alpha (Zarei et al., 2019). Arsenite and MMA^III^ also suppress B cell development from hematopoietic stem cells by altering IL-7 signaling in mice (Ezeh et al., 2014). It has also been reported that non-cytotoxic levels of sodium arsenite (0.25–2 μM) inhibit T cell function by repressing the expression of cyclin D3 and CDC25A thereby causing G1 cell cycle arrest (Morzadec et al., 2012). Arsenic also induces T cell apoptosis via activation of Bcl-2 expression that consequently results in decreased IL-4 release (Qin et al., 2008). Arsenic exposure alters lipopolysaccharide (LPS)-induced inflammation caused by macrophages and monocytes as well. Under these conditions, monocytes mount an elaborate immune response characterized by the enhanced production of proinflammatory cytokines and chemokines (Bourdonnay et al., 2011). Low-level chronic arsenic exposure causes inflammation in the exposed individuals. Interestingly, UPR activation also induces the expression of inflammatory cytokines and Toll-like receptors (TLRs) that may play a role in the onset of cutaneous inflammation. Also, sub-chronic arsenic exposure may activate AIM2 inflammasomes, which in turn augment the secretion of proinflammatory cytokines IL-1β and IL-18 in keratinocytes and the skin of experimental mice (Zhang et al., 2016).

The integrity of the immune system is critical for tumor suppression, and several human malignancies have been attributed to arsenic-impaired immunity (Yu et al., 2018). Multiple studies have revealed that dysregulation of innate and adaptive immunity contributes to arsenic-induced skin carcinogenesis (Huang et al., 2019). In Swiss albino mice, acute exposure to inorganic arsenic induces alterations in the architecture of the thymus and spleen, therefore, compromising their immune physiology. A dose-dependent decrease in the ratio of CD4^+^ to CD8^+^ T cells was observed at higher doses of arsenic. Arsenic exposure leads to reduced thymic function in newborns and a decline in peripheral CD4^+^ T cell function and IL‐2 secretion in adults (Conde et al., 2007). In arsenic‐induced Bowen’s disease, there is a decline in the number and activity of epidermal Langerhans cells and a selective apoptosis of CD4^+^ T cells (Yu et al., 2018). The decreased immune activity of Langerhans cells and CD4^+^ T cells provides a conducive microenvironment for impaired immune surveillance for promoting neoplastic lesions in the skin.

## Human disorders due to arsenic exposure

### Skin pathology

Arsenic-induced skin lesions are one of the early hallmarks of groundwater arsenic poisoning. As previously reviewed by Yoshida et al., multiple epidemiological case-control and cohort studies support a consistent dose-response relationship between arsenic levels in water and skin lesions in humans (Yoshida et al., 2004). Long-term exposure to high levels of arsenic in groundwater may lead to arsenicosis. The symptoms of arsenicosis are different from the symptoms of other heavy metal toxicity. Arsenic exposure leads to distinct palmer and plantar hyperkeratosis (hardened patches of skin), hyperpigmentation (dark spots on the skin), and transverse white bands on the fingernails. The early pigmentary changes including dark brown and raindrop-shaped lesions are followed by arsenical keratosis. A population-based study in Bangladesh showed that the duration of consumption of arsenic-containing water is correlated with hyperpigmentation of the forehead skin in humans (Yajima et al., 2018). However, the mechanism of arsenic-mediated hyperpigmentation in the skin remains rather unclear. Recently, it was reported that the interaction between keratinocytes and melanocytes through endothelin-1 (ET-1) activity is responsible for arsenic-mediated skin hyperpigmentation in hairless mice. It was also found that coexposure to arsenic and ET-1 led to melanocyte proliferation and melanin synthesis (Yajima et al., 2017). Arsenical keratosis is precancerous dermatosis characterized by corn-like, hyperkeratotic papules. Interestingly, abnormal tissue differentiation and diminished expression of β_1_, α_2_β_1_, or α_3_β_1_ integrins have been reported in patients with arsenical keratosis (Lee et al., 2006). A recent study by Zeng et al. found that miR-155e5p regulates the NF-AT1 transcription factor-mediated immunological dysfunction responsible for skin lesions during arsenicosis. Krt1 and Krt10 are biomarkers of arsenic-induced hyperkeratosis, while Krt6c is considered a biomarker of arsenic-induced carcinogenesis (Zeng and Zhang, 2020).

As previously reviewed by Tchounwou et al., oxidative stress, genotoxicity, impaired DNA repair, and dysregulation of protein expression have been reported as the underlying mechanisms of cutaneous manifestations of arsenic toxicity (Tchounwou et al., 2019). Moreover, arsenic induces the accumulation of unfolded proteins in the ER lumen, leading to ER stress in skin keratinocytes (Srivastava et al., 2013). Consequently, cells induce unfolded protein response (UPR) signaling, which results in an adaptive augmentation of chaperone protein expression. The chaperone proteins bind to unfolded polypeptides until they are properly folded, thereby reducing the ER stress (Weng et al., 2014). In addition, the transcriptional activation of UPR genes results in global translational attenuation and ER-associated protein degradation to maintain the protein-folding homeostasis (Grootjans et al., 2016). During ER stress, the chaperone GRP78/BiP dissociates from ER membrane sensors like IRE1α, PERK, and ATF6 resulting in their phosphorylation and activation. Activated IRE1α splices X box-binding protein 1 (XBP-1) and eventually leads to activation of UPR target genes in the skin (Li et al., 2011). The C/EBP homologous protein CHOP plays an essential role in arsenic-induced ER stress, and its expression is regulated by the transcription factors ATF4 and ATF6. Thus we found that ATO induces CHOP activation and cellular apoptosis through the PERK/ATF4-dependent pathway (Srivastava et al., 2016).

Chronic exposure to arsenic through drinking water has been associated with an increased risk of skin cancer in addition to cancers of the lung, bladder, and kidney in humans. Arsenic-induced skin cancers in humans include squamous cell carcinoma (SCC), basal cell carcinoma (BCC), and Bowen’s disease (in situ carcinoma). Numerous studies have indicated that biomethylation converts inorganic arsenic into carcinogenic metabolites (Kojima et al., 2009). Various mechanisms, including oxidative stress, DNA damage, chromosomal aberrations, epigenetic modifications, and immune dysregulation have been previously associated with arsenic-induced skin cancers (Martinez et al., 2011). Emerging research reveals that arsenic may trigger the generation of cancer stem cells (CSCs) from normal epithelial stem cells (Tokar et al., 2010). Waalkes et al. also demonstrated that arsenic leads to malignant transformation of stem cells (Waalkes et al., 2008). As reviewed by Li et al., arsenic exposure in mice might convert embryonic stem cells or keratinocyte stem cells into the CD34+ cancer stem cells. In addition to the existing stem cells, chronic arsenic exposure may induce dedifferentiation of the differentiated cells to generate cancer stem cells (Li et al., 2019).

Arsenic-induced changes in DNA methylation, activation of oncogene expression, changes in tumor suppressor gene expression have been reviewed previously (Reichard and Puga, 2010). Epigenetic alterations play a vital role in the regulation of telomere length, and telomeric DNA integrity is critical for cell survival, genome stability, and malignant transformation (Bernal and Tusell, 2018). Arsenic has been reported to cause telomere lengthening in a telomerase-independent manner (Fig. 2). Indeed, a two-fold telomere length increase was detected in 85% of arsenic-induced skin cancer tissue samples. This was accompanied by modifications in telomeric DNA methylation patterns and depletion of the histone modification H4K20me3 (Bhattacharjee et al., 2020). Bowen’s disease is characterized by multiple recrudescent lesions. This noninvasive intraepidermal SCC has been linked to epigenetic dysregulation, with alterations in the pattern of DNA methylation and histone modifications reported in arsenic-exposed mice and human samples (Bjørklund et al., 2018). Furthermore, cyclin D1 overexpression in arsenic-associated urothelial carcinomas is a result of DNA hypomethylation. Thus unmethylation of cyclin D1 promoter is a likely mechanism for the progression of arsenic-induced Bowen’s disease pathogenesis (Liao et al., 2018). Post-translational modifications of histones such as H3 methylations have also been reported in skin lesions from individuals with chronic arsenic exposure. Specifically, DOT1L methyltransferase-regulated H3K79me1 epigenetic signature was detected in the arsenic exposed humans (Bhattacharjee et al., 2018). Another study reported the changes in the expression of SUV39H2 (H3K9me3 methyltransferase) in arsenic-treated keratinocytes. The SUV39H2-mediated epigenetic changes in the promoter of E2F1 (transcription factor) followed by centrosome amplification suggest a role in arsenic carcinogenesis (Liao et al., 2017).

Regulatory non-coding RNA including long non-coding RNAs (lncRNAs) and miRNA have been reported to play an important role in arsenic-induced carcinogenesis. The expression of oncogenic LncRNA HOTAIR and LncRNA-p21 was increased after chronic arsenic exposure (Tan et al., 2021). A study designed to identify the role of non-coding RNA in arsenic-induced skin carcinogenesis in humans revealed an enhanced miR-21 expression and subsequent activation of the PI3K-AKT cell survival pathway and cancer (Banerjee et al., 2017). Epigenomic miRNAs may play a vital role in the regulation of protooncogenes and tumor suppressor genes. Arsenic-associated premalignant and malignant skin lesions manifest differential expression of 35 miRNAs. Out of these, the expressions of miR-425-5p and miR-433 were induced in both BCC and SCC, whereas miR-184 and miR-576-3p were specifically induced in SCC only (Al-Eryani et al., 2018). However, their mechanistic link to cell cycle regulation and skin tumorigenesis is still lacking.

### Cardiovascular disorders

The epidemiological studies correlating chronic arsenic exposure and cardiovascular diseases have previously been reviewed by Stea et al. (Stea et al., 2014). Evidence from animal and human studies indicates that arsenic exposure during the early stages of life may damage the vascular system. Individuals in early life (age less than 20 years) exposed to arsenic have a significantly higher risk of cardiovascular diseases than those exposed in the later stages of life (Hsieh et al., 2015). Hypertension, carotid atherosclerosis, and ischemic heart disease have been reported in individuals chronically exposed to arsenic (Zhao et al., 2021). Milutinović et al. reviewed the role of endothelial cell activation, proinflammatory cytokine production, and accumulation of oxidized low-density lipoprotein in the early stages of atherosclerosis (Milutinović et al., 2019). Arsenic induces expression of HO1, monocyte chemoattractant protein-1 (MCP-1), and pro-inflammatory IL-6, which lead to migration of monocytes and promote atherosclerosis (Wang et al., 2012). Arsenic induces caspase activation, cardiomyocyte degeneration, and myocardial tissue injury in experimental rats (Xue et al., 2020). A recent study evaluating ultrastructural changes in cardiac tissue in sodium arsenite-fed Wistar rats showed dose-dependent remodeling of cardiac tissue including parenchyma loss, collagen deposition, sarcomere disorganization, and myofilament dissociation (Souza et al., 2020).

The significance of arsenic biomethylation was investigated by Silvia et al. and it was demonstrated that methylated arsenicals, including MMA^V^, MMA^III^, and DMA^V^, are proatherogenic and cause atherosclerotic lesions in mice. Exposure of *apoE*^*−/−*^*/As3mt*^*−/−*^ double-knockout mice to different arsenicals suggested that AS3MT function is directly correlated with the risk of atherosclerosis. The genetic analysis further revealed an association between single nucleotide polymorphisms (SNPs) in the AS3MT gene and cardiovascular disorders (Liu et al., 2020). The AS3MT SNPs that correlate with enzyme function could predict the risk of developing atherosclerosis in arsenic-exposed populations (Negro Silva et al., 2017). miRNAs might also have a role in arsenic-induced cardiovascular diseases. In Mexico, human plasma samples from areas with high arsenic levels in drinking water were analyzed for small RNAs using high-throughput sequencing. These results suggest that circulating miRNAs associated with cardiovascular diseases and diabetes include miR-423-5p and miR-454-5p which are linked to the presence of MMA in plasma (Beck et al., 2018). However, the mere presence of these miRNAs does not establish their direct role in arsenic-induced cardiovascular diseases in humans. Future studies must establish how MMA induces the expression of these small RNAs and whether upregulation of miR-423-5p and other related miRNAs is involved in the degeneration of cardiomyocytes, as also indicated in the previous studies (Luo et al., 2015).

### Diabetes mellitus

The correlation between arsenic toxicity and diabetes mellitus is an emerging public health concern globally. A cohort study of 641 participants from rural Bangladesh showed a dose-dependent correlation between drinking water arsenic levels and the risk of hyperglycemia, impaired glucose tolerance, and diabetes mellitus. This study also revealed an alarming pattern of arsenic-induced hyperglycemia in females than in males (Paul et al., 2019). Another independent study involving 957 adult participants from Bangladesh indicated that exposure to moderate levels of arsenic in drinking water significantly increased the risk of type 2 diabetes (Pan et al., 2013). These observations are not restricted to only one country or geographical region. A recent cohort study in Taiwan reported a significantly higher prevalence of type 2 diabetes among the arsenic-exposed population compared to the general Taiwanese population (Tseng et al., 2000).

Inorganic arsenic has been reported to induce pancreatic β-cell degeneration in animal models via apoptosis, pyroptosis, and ferroptosis (Pei et al., 2019; Wei et al., 2020). Animal studies have revealed that methylated arsenic species, especially DMA^III^ target murine pancreatic islet cells thereby affecting insulin production (Kuo et al., 2017). Beck et al. found that inorganic arsenic leads to downregulation of calcium-dependent protein kinase CAMK2A, involved in insulin secretion and the onset of type 2 diabetes. CAMK2A is a target of regulatory miR-146a, and high-throughput sequencing data show that miR-146a is significantly upregulated by exposure to inorganic arsenic (Beck et al., 2019).

Insulin resistance is also a major concern in arsenic-exposed individuals. A recent human study in US adults revealed a strong correlation between urinary arsenic levels and insulin resistance (Zhou et al., 2022). Inflammation, oxidative stress, and apoptosis contribute to insulin resistance and diabetes. Arsenic may induce proinflammatory cytokines, which may activate the JNK pathway. JNK is known to inhibit the activity of insulin receptors, which in part explains insulin resistance in arsenic-exposed individuals (Lee et al., 2003). Also, chronic exposure to trivalent arsenic species inhibits 3-phosphoinositide-dependent kinase-I (PDK-1) and GLUT4 recruitment to the plasma membrane of insulin-stimulated adipocytes. The activity of PDK-1 is essential for GLUT4 translocation, glucose transport, and storage. This provides a key explanation for arsenic-induced inhibition of insulin-dependent glucose uptake and hyperglycemia. Recently, Li et al. showed that translocation of GLUT4 in hepatocytes is inhibited by arsenite and miR-191 might diminish the translocation of GLUT4 (Li et al., 2021). Although this study shows a promising role of miR-191 in glucose transport and metabolism in human hepatocytes, the in vivo molecular data are still not sufficiently available to draw a definitive conclusion.

### Neurotoxicity

Arsenic has been associated with several neurological disorders, including neurodevelopmental defects, as reviewed by Tyler and Allan (Tyler and Allan, 2014). Based on animal experiments, several underlying mechanisms of arsenic-induced neurotoxicity including mitochondrial dysfunction, apoptotic cell death, and, thiamine deficiency have been proposed. Mitochondrial biogenesis plays a vital role in maintaining normal mitochondrial function. The peroxisome proliferator-activated receptor gamma coactivator-1α (PGC-1α) is the master regulator of mitochondrial biogenesis. Oral sodium arsenite administration (25 ppm) in rats decreased the activity of mitochondrial complexes and led to downregulation of PGC-1α, as well as its downstream targets NRF-1 and NRF-2 in the rat brain (Prakash and Kumar, 2016). This may explain the contributory role of arsenic in mitochondrial dysfunction and neurotoxicity. Arsenic also induces an inflammatory response and apoptosis in microglial cells through activation of the p38 MAP kinase/JNK pathway (Mao et al., 2016). Another important report indicated that arsenic causes thiamine deficiency, which may lead to proinflammatory neuronal disorders such as axonal neuropathy (Mochizuki, 2019). ER stress is also a potential mechanism for arsenic-induced neurotoxicity, as arsenic-induced ER stress triggers UPR signaling. In this regard, miR-124 has been identified as a suppressor of ER stress-induced apoptosis (Panganiban et al., 2019), and a recent study demonstrated the neuroprotective role of miR-124 during arsenic-induced ER stress. Interestingly, genetic polymorphisms of miR-124 are associated with neurocognitive outcomes in children (Park et al., 2020). This study identifies miR-124 as a potential therapeutic target against arsenic-induced ER stress and neurotoxicity. Selenium, an essential trace element with antioxidant functions, has been shown to alleviate arsenic-induced neurotoxicity in rats (Adedara et al., 2020).

Several neurological disorders such as Alzheimer’s disease are associated with protein misfolding and proteotoxic stress in human cells. Although no direct cause-effect relationship has been established so far. However, arsenic exposure causes phosphorylation of tau protein and overexpression of the amyloid precursor protein, which is involved in the formation of brain amyloid plaques (Gong and O’Bryant, 2010). Also, chronic exposure to inorganic arsenic leads to increased amyloid-β production and β-secretase activity in the rat brain (Niño et al., 2017). Arsenite exposure leads to proteotoxic stress via interaction with the RING finger motif in ZNF598, a sensor protein involved in ribosome-associated protein quality control (Tam et al., 2020a). Although several studies associate chronic arsenic toxicity with neurodegenerative diseases, the present molecular data are not yet sufficient to establish this correlation in humans. Key molecular and biochemical data in affected populations are needed to establish this link. Also, it would be interesting to determine the gender-specific risk of arsenic-induced neurodegeneration.

### Nephrotoxicity

Chronic exposure to inorganic arsenic has been associated with multiple nephrological disorders, including chronic kidney disease and cancer. As reviewed by Khairul et al., the levels of inorganic as well methylated arsenic in urine are strongly associated with the incidence of nephrotoxicity. MMA^III^ shows more cytotoxic effects in human urothelial cells than other trivalent arsenicals such as inorganic As^III^ and DMA^III^ (Khairul et al., 2017). Arsenic accumulation in kidneys can cause dysfunction of proximal tubules and kidney fibrosis and may reduce the glomerular filtration rate. Mechanistically, arsenic may induce fibrogenic changes in human kidney cells by promoting epigenetic changes, including DNA methylation patterns (Chang and Singh, 2019b). Kidneys are one of the target organs for arsenic-induced carcinogenesis. Arsenic-induced neoplastic transformation in human kidney cells involves activation of Wnt β-catenin/c-myc pathway (Chang and Singh, 2019a). Arsenic exposure may also lead to nephrocalcinosis, hypercalciuria, and tubular necrosis. Kidney injury molecule 1 (KIM-1) and neutrophil gelatinase-associated lipocalin (NGAL) are highly sensitive biomarkers for detecting arsenic-induced kidney damage (Cárdenas-González et al., 2016).

### Pulmonary diseases

Exposure to moderate levels of arsenic in drinking water is epidemiologically associated with impaired lung function. Obstructive lung disease and bronchiectasis are reported in individuals chronically exposed to groundwater arsenic in Bangladesh (Mazumder, 2007). Metabolic and immune dysfunction may contribute to arsenic-induced pulmonary damage. Chronic exposure to arsenic may also cause pulmonary fibrosis via M2 macrophages. The long non-coding RNA, H19, has been reported to mediate M2 polarization of macrophages and promote myofibroblast differentiation during arsenic-induced pulmonary fibrosis in mice (Xiao et al., 2021). However, it remains to be seen if a similar mechanism is responsible for arsenic-induced pulmonary toxicity and fibrosis in humans. Based on a rural population study in Bangladesh, it was found that individuals consuming arsenic-contaminated drinking water are at a higher risk of developing lung cancer (Mostafa et al., 2008). Another population-based case-control study in 10 counties in two US states found a correlation between toenail arsenic levels of individuals and lung cancer incidences (Heck et al., 2009). Interestingly, Breda et al. revealed that epigenetic modifications such as DNA methylations might underlie the activation of transcription factors associated with tumor progression in arsenic-induced lung cancer (van Breda et al., 2015).

## Arsenicals as chemical warfare agents

The use of arsenicals in chemical warfare is another aspect of arsenic chemistry. The chemical replacement of one or more chlorine atoms in AsCl_3_ by organic moieties leads to highly toxic derivatives. The organoarsenicals synthesized during the first half of the twentieth century include methyldichloroarsine, ethyldichloroarsine, phenyldichloroarsine, and lewisite. Due to their rapid debilitating toxic manifestations and cost-effective synthesis, many of these highly toxic organoarsenicals were produced in large quantities and stockpiled for use in warfare. Arsenical CWAs can be broadly classified as vomiting agents (e.g., adamsite), vesicants (e.g., lewisite), and blood agents (e.g., arsine, inorganic arsenical).

### Arsenical stockpiles and sea-dumped munitions

The large-scale production of lewisite started during WWII in the USA, Germany, Great Britain, Japan, and the former Soviet Union. In the USA, lewisite was developed at a facility in Cleveland, Ohio, and later at Pine Bluff, Arkansas, and Huntsville, Alabama (Pechura and Rall, 1993). Although the USA has destroyed most of its stockpiled CWAs, the remaining stockpiled and non-stockpiled CWAs are still a matter of concern. The Russian Federation is the home of the former Soviet CWA stockpile including lewisite, mustard gas, and mixtures. Large quantities of lewisite are believed to be stockpiled in Kambarka and Gorny regions in Russia (Vilensky and Sinish, 2004).

**Figure 3:**
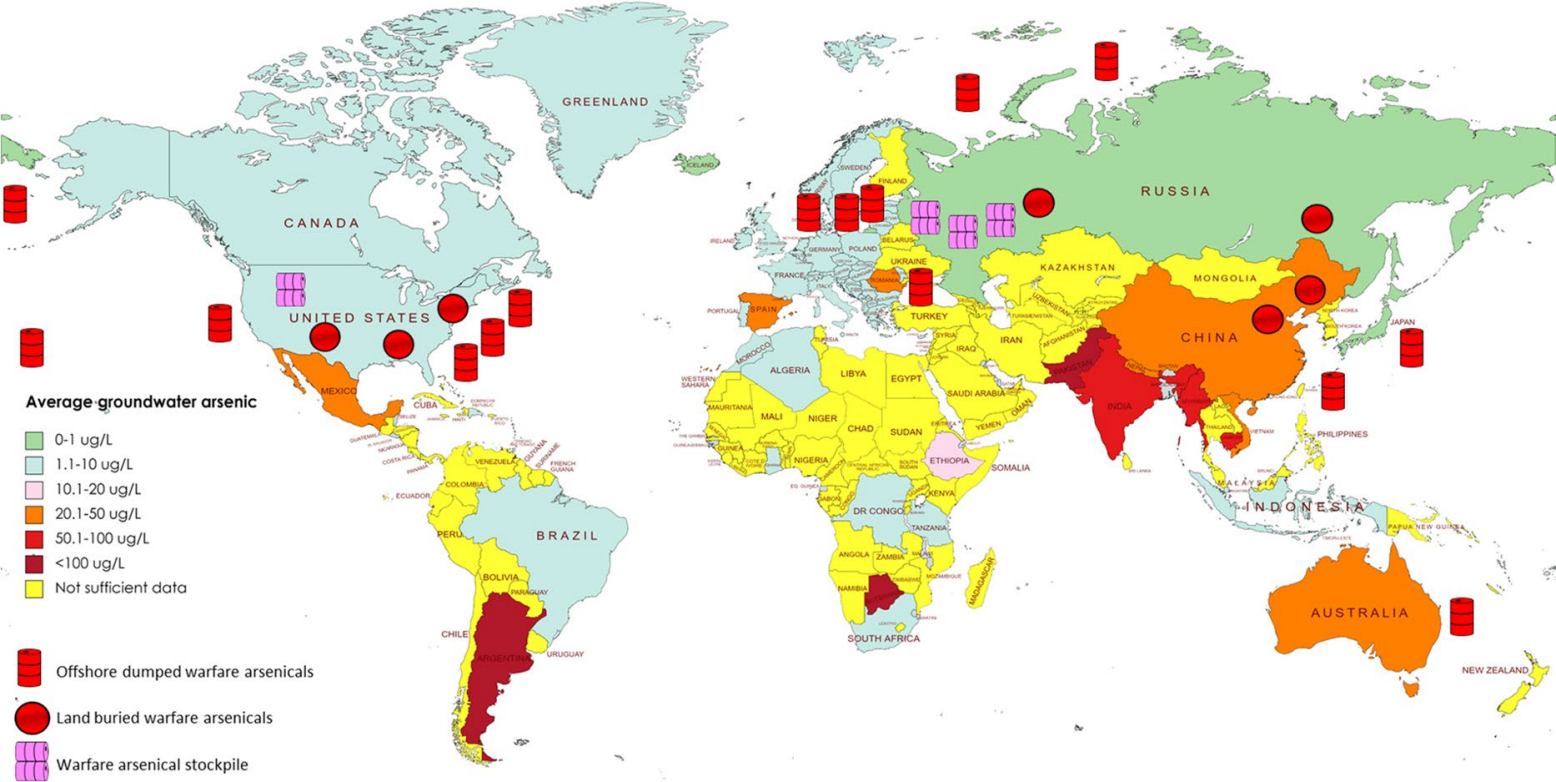
An overlay of global distribution of groundwater arsenic and dumped/buried warfare arsenicals. This map depicts the average groundwater arsenic concentrations in different countries around the world (based on data from Podgorski et al; 2020) as well as the distribution of warfare arsenicals including existing stockpiles, land buried and offshore dumped arsenicals. This figure was prepared using mapchart.net

The massive quantities of CWAs produced during WWI and WWII were mostly unused and the Potsdam conference in 1945 resolved that CWAs would be dumped into the basins of the Baltic Sea.. Thereafter, it became a worldwide practice to dump CWAs and military equipment into the ocean or to bury them (Fig. 3). Moreover, considering it as the cheapest method of disposal, the USA, USSR, and UK dumped their chemical weapons directly into various oceans. According to the Congressional Research Service (CRS) report, the U.S. Army cataloged 74 instances of CWA disposal, which included 32 cases off the US coastline and 42 instances off foreign shores (Bearden, 2006). The CWAs dumped off the US coastline included organoarsenical CWAs, as summarized in Table 2. These offshore dumping sites included the coasts of the Atlantic Ocean, Pacific Ocean, the Gulf of Mexico, and the coast of Hawaii. During WWII, the USA had stockpiled CWAs including lewisite and adamsite in other countries including Australia. In 1946, most of these stockpiles were dumped off the coast of Queensland (Vilensky, 2005). The Japanese administration declared several offshore dumping sites including the Pacific Ocean as well as Japanese rivers and lakes. These offshore dumped CWAs included organoarsenicals including lewisite, Clark I, and Clark II (Radke et al., 2014). Although the total amount of lewisite produced by the Soviet Union is not known, around 132,000 tons of lewisite were dumped into the Arctic Sea during the 1940s and 1950s (Vilensky and Sinish, 2004). In Europe, most of the CWAs were dumped in the Baltic Sea and the Skagerrak Strait. The quantity of some of the organoarsenical CWAs dumped in the Bornholm Basin in the Baltic Sea has been reported in the CWA report by the Danish Centre for Environment and Energy (Table 3).

**Table 2:** Dumping sites of arsenic-based CWA around the US coastline. This table is an indicative of the magnitude of potential risk associated with accidental exposure to offshore dumped arsenical. (Prepared based on Bearden et al. 2006)

Year	Disposal site	Origin of chemical agents	Chemical agents
1945	Atlantic Ocean, “Disposal Area Number 1”	Edgewood Arsenal, Maryland	• 1154 drums (55 gal) of arsenic trichloride
			• 375 tons of adamsite
1945	Pacific Ocean, off Hawaii	Waianae, Hawaii	• 190 1-ton containers of lewisite
1946	Atlantic Ocean, “Baker” Site off Charleston, South Carolina	-	• Lewisite (quantities unspecified)
1947	Pacific Ocean, 12 miles off Aleutian Islands	Attu and Adak, Alaska	• 887 containers of lewisite
1948	Atlantic Ocean, 300 miles off Florida	Gulf Chemical Warfare Depot, Alabama	• 3711 containers of lewisite
1957	Atlantic Ocean	Edgewood Arsenal, Maryland	• 48 1-ton containers of lewisite
1958	Atlantic Ocean, off South Carolina	Pine Bluff Arsenal, Arkansas	• 1507 1-ton containers of lewisite
1958	Pacific Ocean, 117 miles off San Francisco, California	Navajo Army Depot, Arizona, and Tooele Army Depot, Utah	• 1479 1-ton containers of lewisite
1958	Pacific Ocean, 117 miles off San Francisco, California	Tooele Army Depot, Utah	• 335 1-ton containers of lewisite
1960	Atlantic Ocean	Edgewood Arsenal, Maryland	• 2 1-ton containers of lewisite
			• 1 lewisite cylinder
1962	Atlantic Ocean	Edgewood Arsenal, Maryland	• 1 1-ton container of lewisite
1968	Atlantic Ocean	Colts Neck Naval Pier, Earle, New Jersey	• 120 drums of canisters of arsenic and cyanide

**Table 3:** Confirmed amount of dumped CWAs in Bornholm Basin in the Baltic Se. (These data were extracted from the CWA report by the Danish Centre for Environment and Energy, Sanderson et al. 2015)

Compound	Amount dumped (tons)	Water solubility (mg/L)
Adamsite	1428	0.4
Clark I	711.5	3
Triphenylarsine	101.5	0.089
Phenyldichloroarsine	1017	639
Trichloroarsine	101.5	2291
Lewisite	Unknown	0.5

The European chemical munitions projects such as MERCW and CHEMSEA, along with the NATO SPS Project MODUM, have located munitions in the Baltic Sea and Skagerrak areas (Niemikoski et al., 2017). It was revealed that some of these CWA containers are disintegrating and their contents are leaking (Briggs et al., 2016). The CHEMSEA project involved analysis of phenylarsenic CWAs from the sediment collected from the Bornholm Basin deep in the Baltic Sea. Gas chromatography-tandem mass spectrometry (GC-MS/MS) analysis showed the highest detected arsenical concentrations of 1300, 210, and 41 µg/kg dried sediment, respectively, for Clark I, adamsite, and phenyldichloroarsine-related compounds (Niemikoski et al., 2017). The CHEMSEA project also focused on the chemical analysis of marine biota exposed to arsenicals. In 2002, independent studies carried out by The Norwegian Defence Research Establishment showed that ammunition dumped in the Skagerrak Strait was corroded and leaking CWAs. The sediment samples collected from these sites showed significantly high concentrations of Clark 1, triphenylarsine, and bis(diphenylarsinic)oxide (BDPAO). The highest concentrations of Clark I, BDPAO, and triphenylarsine were recorded as 178, 137, and 63 mg/kg of sediment respectively (Tørnes et al., 2002). Due to the CWA degradation products, there is a serious risk of direct or indirect human exposure. Czub, M., et al. demonstrated the adverse effects of organoarsenic CWAs on fish biodiversity and ecosystem around the CWA dumping sites in the Bornholm Deep area (Czub et al., 2018). The toxic effects of these organoarsenical CWAs on humans via contaminated fish consumption are largely unclear. However, a study by Sanderson et al. provided a predictive model-based assessment of the human health risks due to fish consumption from the Bornholm Basin. This qualitative screening revealed the mutagenic and carcinogenic properties of organoarsenic CWAs (Sanderson et al., 2009). More exhaustive research based on long-term implications of consumption of organoarsenic CWAs contaminated seafood on humans is needed to provide definitive insights related to human health impact. Currently, such information is not available.

Land burial has been another common method of CWA disposal in the USA and other countries. Multiple sites in the USA are known or suspected to have buried CWAs including Spring Valley in Washington, D.C., and Redstone Arsenal (Huntsville Arsenal) in Alabama. Redstone Arsenal in Alabama manufactured and packaged CWAs such as lewisite and adamsite from 1940 until 1945. After the war, large quantities CWAs were buried in trenches as a disposal strategy. Although some of the buried CWAs have been decontaminated, the remediation of remaining CWAs and the contaminated material may take years and a huge expenditure (Council, 2012). After the collapse of the Soviet Union, the toxic CWAs were buried in the ground. Today, Dzerzhinsk remains one of the most polluted cities due to scattered contaminated waste and seeping waste from landfills (PURY, 2019). Between 1942 and 1943, Japan’s imperial forces used colossal quantities of vomiting agents and blistering agents (Lewisite and Sulfur mustard) on Chinese populations. After WWII, Japan abandoned large quantities of chemical munitions in China and an estimated 2000 deaths have resulted from accidental exposure to these munitions (Vilensky and Sinish, 2004). Many of the munitions containing lewisite, Clark 1, and Clark 2 have been found scattered or buried all over China (Brombach, 2011). In August 2003, 44 victims were accidentally poisoned by sulfur mustard and lewisite leaked from five drums that were excavated at a construction site in China. The exposed victims largely manifested severe autonomic failure, including hyperhidrosis, pollakiuria, memory loss, and visuospatial disability (Isono et al., 2018).

### Vomiting agents

Vomiting agents were developed as chemical weapons to cause overwhelming sneezing, nausea, vomiting, and bodily discomfort. The major arsenic-containing vomiting agents include Clark I, Clark II, and adamsite. These water-insoluble chemicals can cause irritation of the respiratory tract and eyes. Clark 1 can lead to death if exposure occurs in unventilated, confined spaces (Ochi et al., 2004). However, the toxicity of these agents is comparatively lower than that of vesicants. In aqueous solutions, Clark 1 gradually degrades into diphenylarsinous acid (DPAA), which may further oxidize to diphenylarsinic acid. In Kamisu, Japan, the consumption of water contaminated with the byproducts of Clark I, Clark II such as DPAA has been reported to cause developmental abnormalities, brain atrophy, and cerebral symptoms in humans (Ishii et al., 2004). Adamsite was developed as a riot-control agent during WWI. It was produced and stockpiled by the British and the US armies during WWI (Radke et al., 2014). Inhalation of adamsite or direct exposure to the skin and eyes causes severe vomiting, which compels soldiers to remove their gas masks and therefore expose themselves to other warfare agents mixed with it (Greaves and Hunt, 2011). Along with other CWAs, most of the adamsite stockpiles were dumped into water bodies around Europe. The metabolism and mechanism of adamsite toxicity in humans and marine biota are unclear and the available data is very limited. However, it has been reported that phenylarsenic compounds including adamsite are metabolized by the liver enzymes in the exposed fish. Specifically, adamsite is oxidized, methylated, and glutathione (GSH) conjugated by the enzymes in the cod liver, although the fate and toxicological impact of these metabolites are largely unknown (Niemikoski et al., 2020). Also, adamsite gradually hydrolyzes into phenoarsazin-10(5H)-ol which is very persistent and can bioaccumulate in marine biota (HELCOM). Baltic blue mussels bioaccumulate the oxidized forms of adamsite into their tissues and are therefore considered as bioindicators of organoarsenicals in ocean beds. The adverse effects of the bioaccumulated adamsite derivatives in these mussels include cytotoxic and immunotoxic effects (Höher et al., 2019).

### Blood agents

Blood agents are systemic poisons that affect the body after being absorbed into the blood. Exposure to blood agents causes multiorgan damage, particularly cardiovascular, respiratory, and central nervous system damage (Zilker, 2005). Arsine gas is an arsenic-based blood agent. Being colorless, nonirritating gas, and significantly denser than normal air makes arsine gas a suitable CWA. Arsine gas is also used in the semiconductor industry for material deposition and other manufacturing processes. Accidental release of the gas during the manufacturing processes can expose workers. Arsine gas is the most toxic form of arsenic and inhalation of more than 10 ppm can be lethal (Kuivenhoven and Mason, 2019). It inflicts injury to the respiratory tract due to its direct contact with the lung tissues. The early symptoms of arsine gas toxicity are vomiting, tachycardia, chest pain, headache, fever, and renal failure. Almost 25% of fatal exposure cases reported are due to hemolysis of red blood cells and subsequent kidney failure (Kato et al., 2014). The molecular mechanism underlying severe arsine toxicity is not completely understood. Studies with hairless mice demonstrated that arsine is not absorbed through skin and therefore inhalation remains the major mode of arsine exposure (Kato et al., 2014). Acute arsine poisoning leads to serious oxidative damage to red blood cells by generating hydrogen peroxide and oxyhemoglobin adducts. This leads to rapid hemolysis, renal failure, and sometimes death (Pakulska and Czerczak, 2006). There are no known antidotes against arsine gas toxicity, and chelation therapy like British anti-lewisite (BAL) remains mostly ineffective (Kuivenhoven and Mason, 2019). Therefore, exchange transfusion and dialysis are the only options available to prevent rapid intravascular hemolysis and renal failure (Danielson et al., 2006). In a case report, a 46-year-old man was accidentally exposed to arsine gas during an industrial process. The RBC exchange transfusion by continuous-flow method revealed dark and hemolyzed plasma. Several RBC and plasma exchanges were needed for the recovery of the renal function (Danielson et al., 2006).

### Vesicants

Organoarsenical vesicants that cause blistering skin lesions and induce a severe painful inflammatory response include lewisite, methyldichloroarsine, ethyldichloroarsine, and phenyldichloroarsine. Lewisite was first synthesized in 1904 by Julius Arthur Nieuwland at The Catholic University of America in Washington, D.C., by a reaction of arsenic trichloride with acetylene. It exists in three homologous forms, namely L1, L2, and L3, and the L1 isomer is usually the predominant form. Being highly reactive, lewisite is unstable in the environment due to its hydrolysis to relatively less toxic forms like 2-chlorovinylarsonous acid (CVAA), and 2-chlorovinylarsonic acid (CVAOA). Interestingly, CVAA is also identified as the primary metabolite of lewisite in exposed animals, where it can also be detected in urine (Stanelle et al., 2010). Upon contact with skin, lewisite is readily absorbed within minutes, causing stinging pain, and fluid-filled blisters, especially on the extremities, back, and genitals. Although lewisite is a highly potent vesicant, the molecular mechanism of its pathogenesis is still poorly understood. Previously, our studies demonstrated that topically administered lewisite incites an acute inflammatory response and microvesication in the skin of experimental mice. Oxidative stress, UPR signaling activation, and apoptosis induction in the epidermal keratinocytes characterize the molecular pathogenesis of these cutaneous lesions. Attenuating UPR signaling and oxidative stress in lewisite-challenged animals significantly reduced immediate toxic manifestations (Li et al., 2016). Lewisite induces epidermal-dermal separation in the region of lamina lucida, a component of the basement membrane located between the epithelium and connective tissue. It has been reported that laminin, (a cysteine-rich glycoprotein) is a potential target for lewisite during vesication (King et al., 1994). Lewisite also targets energy metabolism in cells. Glucose consumption in lewisite-treated keratinocytes was diminished to less than 50% within 1 h after exposure (Kehe et al., 2001).

Lewisite toxicity is not only limited to skin, lungs, and eyes. It also causes significant systemic damage to other organs as well. Recently, Manzoor et al. showed that cutaneous lewisite exposure in the murine model leads to acute lung injury as evidenced by increased expression of damage-associated molecular pattern (DAMP) molecule HMGB1 as well as CXCL1 and CXCL5 chemokines (Manzoor et al., 2020). Vascular damage, pulmonary edema, and low blood pressure have also been reported. Lewisite-induced vascular damage is mainly responsible for tissue perforation and hemorrhaging. Depending on the dose of lewisite, the systemic injury can also ultimately result in death (Flora et al., 2009). Similarly, acute kidney injury associated with tubular cell apoptosis, increase in the levels of serum creatinine, and the upregulation of the kidney injury markers KIM-1 and NGAL has been observed in mice treated topically with lewisite (Srivastava et al., 2018). Similar to the results in the skin, lewisite induces ER stress in kidneys, as indicated by the upregulation of ATF4 and CHOP (Srivastava et al., 2020). Eyes are also highly sensitive to lewisite exposure, and lewisite-induced ocular injury is characterized by edema and blepharospasm (abnormal eyelid muscle contraction). It can result in ocular tissue necrosis and eventual blindness (Goswami et al., 2016). Lewisite-induced eye injuries can become irreversible if not treated within a few minutes after exposure.

Medical treatment of lewisite exposure includes BAL (2,3-dimercapto-1-propanol) which shows some efficacy if administered soon after the exposure. BAL is a chelator-type antidote that forms a non-toxic complex with lewisite (Sahu et al., 2013). The topical application of BAL demonstrates some protective effects against lewisite-induced skin lesions in mice. Due to the narrow therapeutic range of BAL, the development of novel lewisite antidotes is needed. However, due to the limited knowledge of the molecular mechanism underlying the pathogenesis of lewisite and the lack of suitable animal models that can recapitulate the molecular pathogenesis in humans, efficacious mechanism-based therapy could not be developed.

## Pharmacological chaperones as therapeutic agents

Despite long known clinical manifestations of arsenic poisoning, the available treatment options are limited. Chelating agents such as BAL, DMPS (dimercapto-propanesulfonate), and DMSA (dimercaptosuccinic acid) have a limited benefit against chronic arsenic toxicity. Demonstration that protein misfolding and aggregation are associated with arsenic toxicity points to the development of novel and effective chemical chaperones that could provide rapid therapeutic benefits. Earlier, misfolded proteins have also been associated with some human pathological conditions known as conformational diseases. Hsp70s and Hsp90s are examples of molecular chaperones that facilitate the proper folding of unfolded and misfolded polypeptides (Genest et al., 2019). Similar to molecular chaperone proteins, chemical chaperones (also known as pharmacological chaperones) can facilitate the renaturation of misfolded proteins and recover their biological activities (Convertino et al., 2016). Although the exact mechanism of action of these agents is still largely undefined, several chemical chaperones can rectify some pathological outcomes especially in animal models (Cortez and Sim, 2014). Sodium phenylbutyrate is known to stabilize the mutant cystic fibrosis transmembrane conductance regulator (CFTR) protein in the ER and circumvent the phenotype associated with destabilizing mutations in cystic fibrosis (Dunmore et al., 2020). Similarly, 4-PBA could be a good therapeutic candidate to circumvent arsenic and arsenical-induced protein misfolding and cellular toxicity. We investigated the impact of chemical chaperone treatment on the attenuation of arsenical-induced cutaneous and systemic injury. These studies revealed an initial evidence that 4-PBA effectively blocks UPR signaling and protects mice against lewisite-induced acute cutaneous inflammation and tissue injury in mice (Li et al., 2016). The allosteric chemical chaperone NAC, which is known to improve the stability of functional proteins without disrupting the catalytic activity (Porto et al., 2012), also blocked UPR signaling and attenuated the lewisite skin injury in mice. It also improved the chaperone activity of 4-PBA (Li et al., 2016). These studies suggest that 4-PBA and NAC are potential candidates for the development of therapeutic interventions against arsenic toxicity. Further investigations are in progress to uncover their mechanism of action.

## Concluding remarks

Arsenic toxicity is a well-known global health concern affecting millions of people worldwide. Cellular stress, enzyme inactivation, and impaired cell metabolism are characteristic features of arsenic exposure both in humans and experimental animals. Chronic exposure to high levels of inorganic arsenic is associated with various human disorders and enhanced susceptibility to multiple diseases. Synthetic organic derivatives of arsenic are extremely toxic and have therefore been used as CWAs. Their environmental and human health effects add another dimension to arsenic toxicity. Most of these unused CWAs were either stockpiled or buried or dumped into various water bodies, including the Baltic Sea, Pacific Ocean, and Atlantic Ocean after WWII, which are still a constant threat to human health and the environment. Their illegal use or accidental exposure provides a rationale for investigating and developing effective antidotes against these agents. Differences in population-based alterations in the toxic manifestations of environmental arsenic, if discovered, may provide a genetic basis for arsenic-related disparities in diagnosis and treatment of the symptoms in exposed populations.

## Data Availability

No datasets were generated during this study.
